# Activity of Free and Liposome-Encapsulated Essential Oil from *Lavandula angustifolia* against Persister-Derived Biofilm of *Candida auris*

**DOI:** 10.3390/antibiotics11010026

**Published:** 2021-12-27

**Authors:** Elisabetta de Alteriis, Angela Maione, Annarita Falanga, Rosa Bellavita, Stefania Galdiero, Luisa Albarano, Maria Michela Salvatore, Emilia Galdiero, Marco Guida

**Affiliations:** 1 Department of Biology, University of Naples ‘Federico II’, Via Cinthia, 80126 Naples, Italy; dealteri@unina.it (E.d.A.); angela.maione@unina.it (A.M.); luisa.albarano@unina.it (L.A.); marco.guida@unina.it (M.G.); 2 Department of Agricultural Science, University of Naples ‘Federico II’, Via dell’ Università 100, 80055 Portici, Italy; annarita.falanga@unina.it; 3 Department of Pharmacy, School of Medicine, University of Naples ‘Federico II’, Via Domenico Montesano 49, 80131 Naples, Italy; rosa.bellavita@unina.it (R.B.); sgaldier@unina.it (S.G.); 4 Department of Chemical Sciences, University of Naples ‘Federico II’, Via Cinthia, 80126 Naples, Italy; mariamichela.salvatore@unina.it; 5 Institute for Sustainable Plant Protection, National Research Council, 80055 Portici, Italy

**Keywords:** *Candida auris*, essential oil, *Lavandula angustifolia*, liposomes, reactive oxygen species, virulence, gene expression

## Abstract

The high virulence of *Candida auris*, a pathogen fungus considered as a global threat for public health, is due to its peculiar traits such as its intrinsic resistance to conventional antifungals. Its biofilm lifestyle certainly promotes the prolonged survival of *C. auris* after disinfection or antifungal treatments. In this work, for the first time, we detected persister cells in a biofilm of *C. auris* in a microwell plate model, following caspofungin treatment. Furthermore, we showed how persisters can progressively develop a new biofilm in situ, mimicking the re-colonization of a surface which may be responsible for recalcitrant infections. Plant-derived compounds, such as essential oils, may represent a valid alternative to combat fungal infections. Here, *Lavandula angustifolia* essential oil, as free or encapsulated in liposomes, was used to eradicate primary and persister-derived biofilms of *C. auris*, confirming the great potential of alternative compounds against emergent fungal pathogens. As in other *Candida* species, the action of essential oils against *C. auris* involves ROS production and affects the expression of some biofilm-related genes.

## 1. Introduction

Since its discovery in 2009 [1], the pathogenic yeast *Candida auris* has elicited several worldwide outbreaks; thus, it is now considered as a global threat to public health, with an estimated mortality range from 28% to 66%. *C. auris* is associated with hospital-acquired infections and clinical isolates generally possess a multidrug-resistant (MDR) profile, with about 30% of isolates showing reduced susceptibility to amphotericin B, and 5% being resistant to echinocandins [2].

Notwithstanding, the phenotypic features of *C. auris* are similar to other *Candida* species; its peculiar thermo- and osmotolerance, together with its intrinsic resistance to conventional antifungals and tolerance to common disinfectants, certainly constitute advantages for spreading and virulence [3,4].

The occurrence of *C. auris* on different medical devices and surfaces has been reported [5,6] to be probably associated with the formation of cell aggregates and biofilms. The peculiar trait of cellular aggregation of some strains [7] and the expression of biofilm-like characteristics can facilitate the prolonged survival of *C. auris* after disinfection or antifungal treatments. *C. auris* sessile cells display increased resistance to antifungals [5,7,8]. Overall, *Candida* spp. cells embedded in biofilms display reduced drug susceptibility with respect to the planktonic counterparts [9].

Several mechanisms are responsible for biofilm resistance, including, production of extracellular biofilm matrix, high cell density, reduction in growth rate, upregulation of drug efflux pumps, stress responses, and the occurrence of persister cells [10,11,12].

Persisters are phenotipic variants which are not susceptible to antimicrobials; they remain alive following treatment, and once the antimicrobial concentration decreases, are able to divide and re-populate the biofilm. Recalcitrant candidemias have been associated with the occurrence of persisters [11]. Persisters have been detected in biofilms of different *Candida* species [10,13] and represent a small fraction of the sessile population in the biofilm. Up to now, persisters have not been described in *C. auris* biofilms. 

In this work, we detected persisters in a *C. auris* biofilm in an in vitro model, following treatment with increasing concentrations of caspofungin. We chose caspofungin since it is the most active drug against *C. auris* infections [14]. Then, we followed the development of a new biofilm of *C. auris* derived from persisters in the microwell plate model and investigated the possibility to eradicate it by using the essential oil from *Lavandula angustifolia*.

In the context of new strategies to face the threat of resistant microorganisms, essential oils (EOs) represent a valid alternative in the treatment of fungal infections and as modulators of fungal biofilms [15,16]. Even if plant-derived compounds have become an important potential for counteracting therapeutic failures in the treatment of fungal infections, literature data on their action against emerging yeast pathogens are still limited [17]. The potential of both bark and leaf *Cinnamomum zeylanicum* EOs to exert antifungal activity against *C. auris* has been reported, showing their ability to damage the membrane structures of fungal cells [18], but the treatment of persistent infections remains a challenge, and the activity of EOs against *C. auris* biofilm has not yet been studied.

Lavender is one of the most commonly cultivated plants in the world, belonging to the *Lamiaceae* family, and the most common species are: *L. angustifolia* Mill (narrow-leaved lavender, usually medical), *L. stoechas* (French lavender), *L. latifolia*, and their hybrids [19]. Lavender EOs showed antiviral activity against *Herpes simplex* virus type 1 [20], antibacterial activity against *Staphylococcus aureus* (MRSA, Methicillin Resistant Staphylococcus Aureus) resistant to methicillin or against *Enterococcus* sp. vancomycin-resistant strains [21]. *L. angustifolia* Mill EO was more effective towards Gram-negative bacteria from the *Enterobacteriaceae* family than Gram-positive *S. aureus* bacteria [22].

Liposomes are spherical lipid vesicles made of one or more lipids that serve as carriers for hydrophilic, lipophilic and amphiphilic compounds. They have been widely used to encapsulate several drugs, since they protect drugs from undesired metabolic breakdown, increase their accumulation at the target site and reduce their toxicity. Encapsulation of EOs in liposome represents an established strategy to overcome some limitations in the use of EOs, regarding their stability towards oxygen, light and temperature, decreases the evaporation or the transfer rate to the outside environment. Liposome encapsulation also enhances EOs solubility and enhances dilution to achieve a uniform distribution in the final product and consequently bioavailability [23]. There are some disadvantages as the high production cost, the chances of leakage of encapsulated compounds, and the presence of phospholipids as liposome constituent that could undergo themselves to oxidation and hydrolysis reaction [24,25,26].

In this study, we encapsulated the EO from *L. angustifolia* in liposomes and characterized them through dynamic light scattering in order to determine the size and the zeta potential and tested their eradication activity towards *C. auris* persister-derived biofilm. Furthermore, some insights into the working mechanisms of action of both free and encapsulated oil on *C. auris* cells in the persister-derived biofilm were provided, by analysing the redox status and the transcriptional expression of some biofilm-related genes (*ALS5, CDR1, ERG11, HOG1*).

## 2. Results

### 2.1. Detection of Persisters Cells in C. auris Biofilms

**Figure 1:**
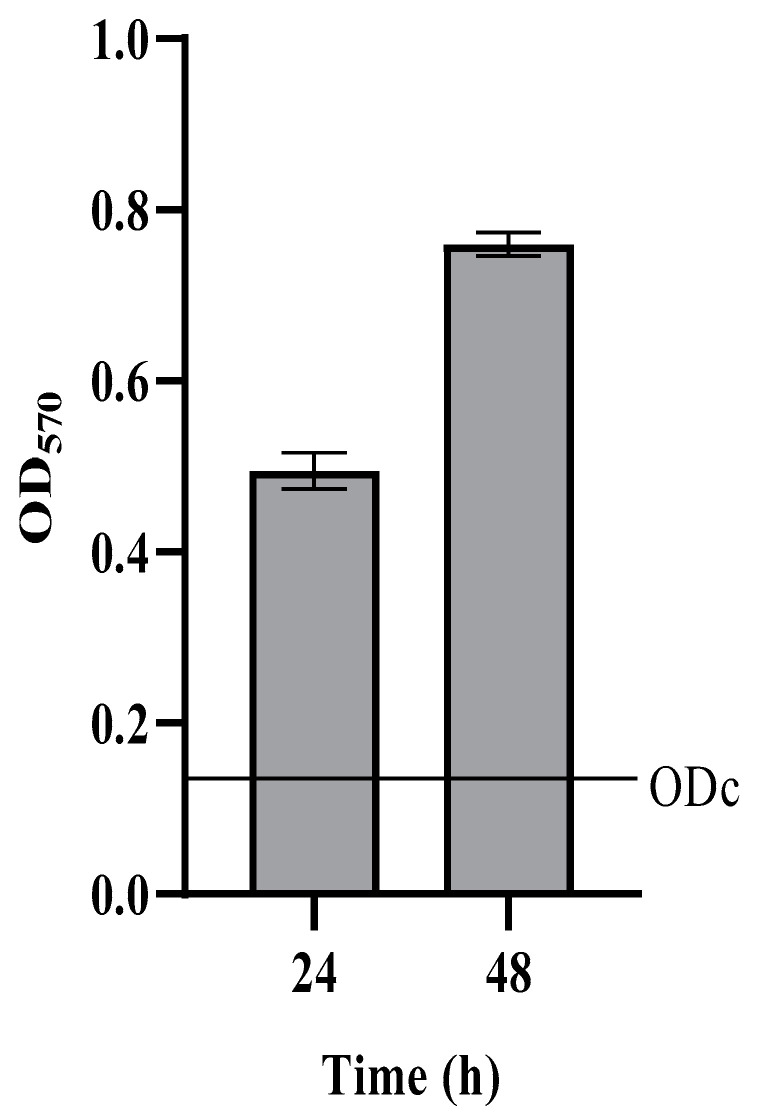
Total biomass of *C. auris* biofilms after 24 and 48 h of incubation. ODc = optical density cut off value (for details see Section 4). Data are the mean of three independent experiments ± SD.

*Candida auris* DSM 21092 was able to form strong biofilms in the in vitro model of the microwell polystirene plate (Figure 1). Total biomass of the sessile population increased between 24 and 48 h, showing that a more mature biofilm was formed at the end of the incubation period.

**Figure 2:**
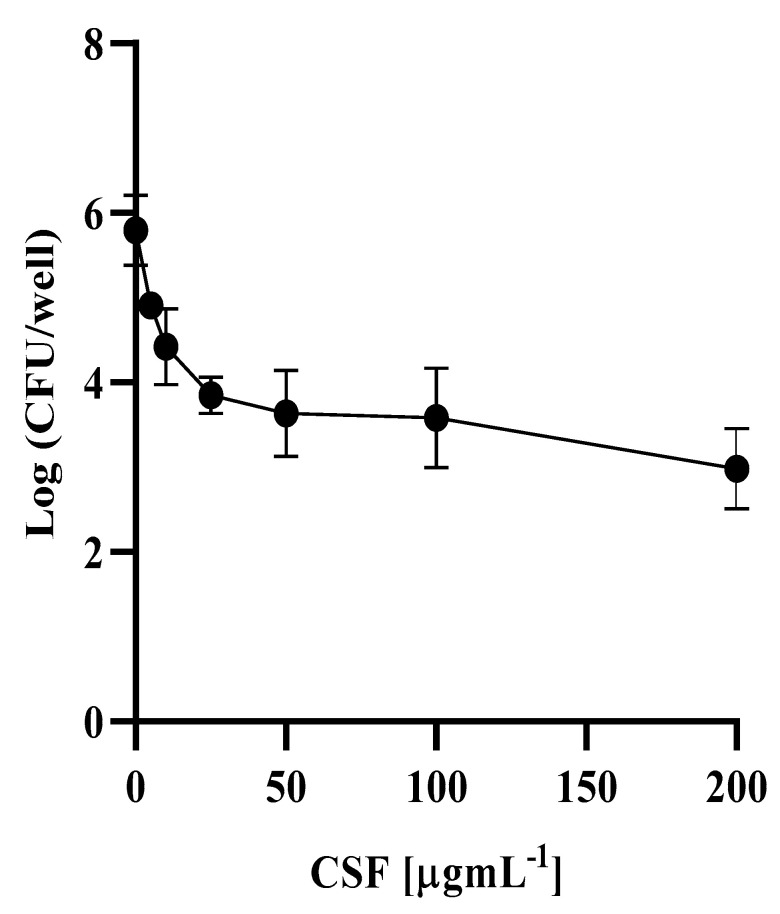
Detection of persisters in *Candida auris* biofilm: survival of *C. auris* cells in biofilm following treatment with caspofungin (CSF) at different concentrations. Data are the mean of three independent experiments ± SD.

Once established on the polystirene surface, the 48 h *C. auris* biofilm was treated with increasing caspofungin concentrations up to 200 × MIC, with an MIC value of the examined *C. auris* strain being 1 ± 0.2 μg mL^−1^. Due to caspofungin treatment, a dose-dependent killing of the biofilm population was observed (Figure 2).

The biphasic-killing curve revealed that a subpopulation of cells in the biofilm was not susceptible, even to the highest antifungal concentration used.

According to similar curves reported for biofilms of several *Candida* spp. subjected to an antifungal challenge, also in the case of *C. auris*, the phenotypic tolerant variants constitute the so-called persisters, representing a very small fraction (0.05%) of the entire sessile population.

**Figure 3:**
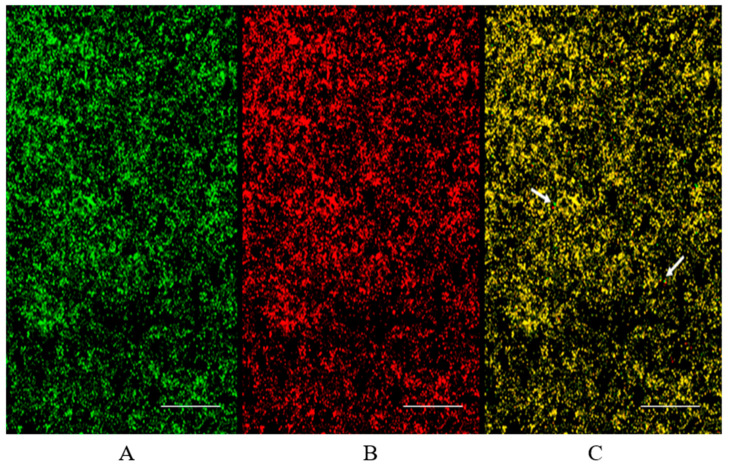
Visualization at CSLM of persister cells in *C. auris* biofilm treated with 200 µg mL^−1^ of caspofungin and stained with SYTO9/PI: (**A**) observation at SYTO (9 λ_max_: all cells in the biofilm appear green; (**B**) observation at PI λ_max_: cells in the biofilm are not alive and appear red; (**C**) merge: very few cells appear bright green (persisters). Arrows indicate the few persisters. Bars correspond to 50 µm.

Live persister cells in the biofilm could be observed by Confocal Laser Scanning Microscopy (CLSM), after staining with Syto9 and propidium iodide (PI) (Figure 3). Indeed, since SYTO9 and PI differ in their ability to penetrate healthy cells, the use of the mixture SYTO9/PI was revealed to be useful in identifying persisters cells in *C. auris* biofilm population treated with 200 mg mL^−1^ caspofungin, where the very few live persisters appear bright green (Figure 3).

**Figure 4:**
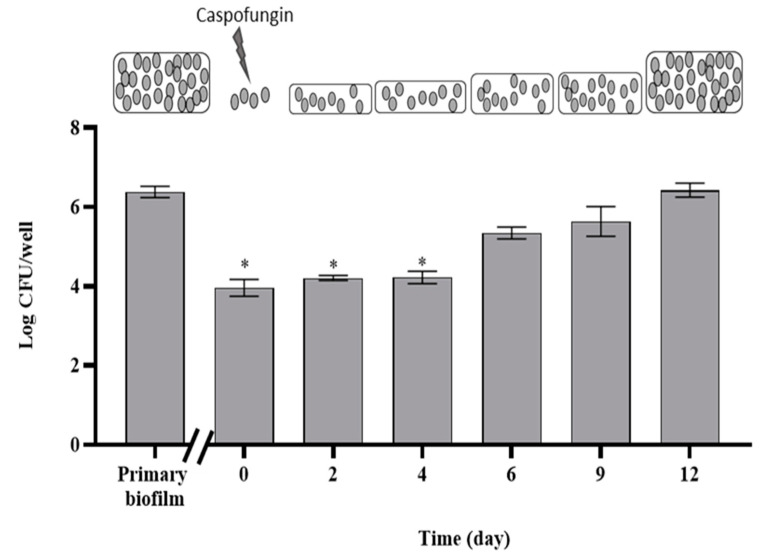
Development of a new biofilm from persisters. Viable cells increase during development of the biofilm derived from cells of the primary biofilm survived at caspofungin treatment (persisters) at time = 0. On the top, a schematic representation of the development of the persister-derived biofilm. * represent significant difference vs. primary biofilm, *p* < 0.05 (Holm–Sidak’s test).

When fresh medium was dispensed into the wells of a microplate where a *C. auris* biofilm (primary biofilm) had been challenged with 200 µg mL^−1^ caspofungin, a new biofilm progressively developed starting from the residual living cells (persisters). Indeed, the number of living cells in the persister-derived biofilm increased over time, achieving the same value as that of the primary biofilm after 12 d (Figure 4).

### 2.2. Composition of L. angustifolia Oil

According to GC-MS analysis, the following major constituents, as shown in Table 1, (peak area percent > 0.5), were identified in the *L. angustifolia* oil ordered upon increasing retention index (RI): 1,8-cineole (1.20%), β-pinene (1.29%), 4-terpineol (1.40%), β-farnesene (1.77%), caryophyllene (2.01%), lavandulyl acetate (2.17%), linalool (39.31%) and linalyl acetate (48.45%). The main constituents were linalyl acetate and linalool.

### 2.3. Characterization of Liposome-Encapsulated Oil of L. angustifolia

Liposomes loaded with oil of *L. angustifolia* were characterized. The hydrodynamic diameter (DH) and polydispersity index (PDI) were measured using dynamic light scattering (DLS). Three independent experiments were performed for each sample and each measurement was performed at least in triplicate. Liposome solutions present a polydispersity index (PDI) < 0.3 indicating a good size distribution (Table 2).

Moreover, our data clearly indicate that the *L. angustifolia* oil is completely encapsulated in the experimental conditions used in this study. In particular, the ratio between the oil volume and the lipid volume is 0.1, indicating that the encapsulated oil is not able to destroy the liposome packing.

### 2.4. Antifungal Activity of L. angustifolia Free and Encapsulated Oil on Planktonic Cells and Biofilms of C. auris

**Figure 5:**
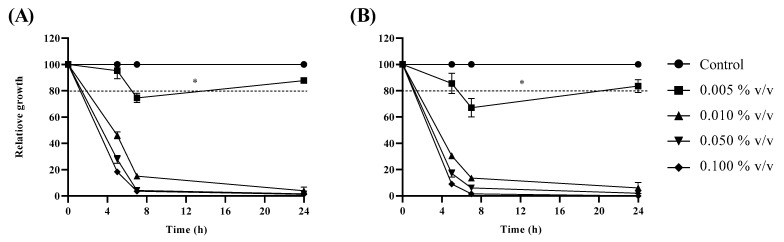
Effect of *L. angustifolia* free (**A**) and liposome-encapsulated oil (**B**) on planktonic *C. auris* growth. Values below the dotted line are statistically significant, * *p* < 0.05 (Holm–Sidak’s test).

**Figure 6:**
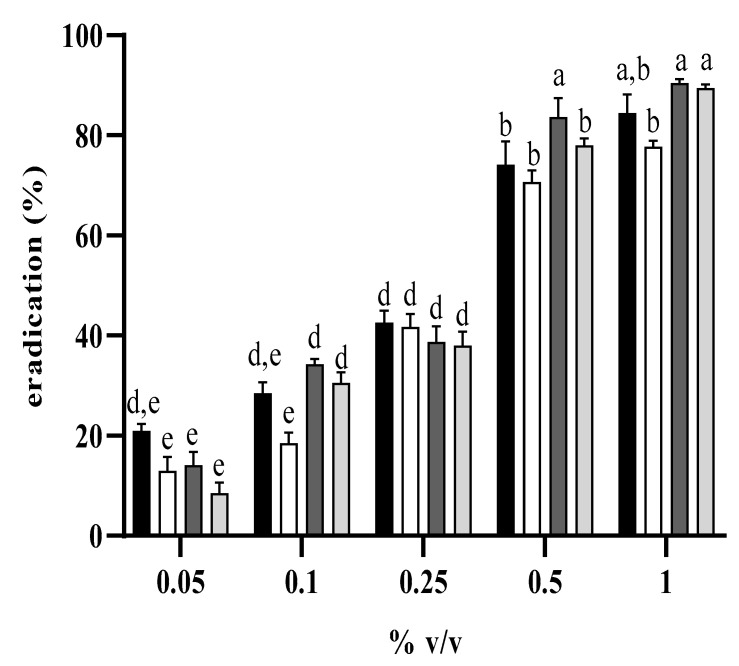
Eradication of free and liposome-encapsulated *L. angustifolia* oil on *C. auris* biofilms. Effect on primary biofilm: free oil (black bars); liposome-encapsulated oil (white bars). Effect on persister-derived biofilm: free oil (dark grey); liposome-encapsulate oil (light grey). Bars with different letters (a,b,d,e) are significantly different, bars with the same letters (a,b,d,e) are not significantly different (Tukay’s test, *p* < 0.05).

When *C. auris* cultures were incubated in the presence of increasing concentrations of either *L. angustifolia* free or liposome-encapsulated oil, growth reduction was monitored during 24 h incubation. In Figure 5A,B, the effect of the examined compounds on planktonic fungal growth is reported; a significative growth reduction was already observed when cells were incubated in the presence 0.01% *v*/*v* free or encapsulated oil, but a slight effect was already visible at a concentration two-fold lower. Both 48 h primary and persister-derived biofilm of *C. auris* were eradicated when treated with *L. angustifolia* free or encapsulated oil, in a concentration dependent manner (Figure 6). Eradication of biofilms was obtained at concentrations higher than those determining planktonic growth reduction, in accordance with the general lower susceptibility of biofilms to antimicrobial compounds. Furthermore, no significant difference in biofilm eradication was observed between the two oil formulations, with a value of more than 80% eradication achieved at a concentration of 0.5% *v*/*v* (Figure 6). No eradication at all was detected by using the empty liposome (data not shown), showing that the eradication effect was due to the encapsulated oil, and not to the liposome components.

**Figure 7:**
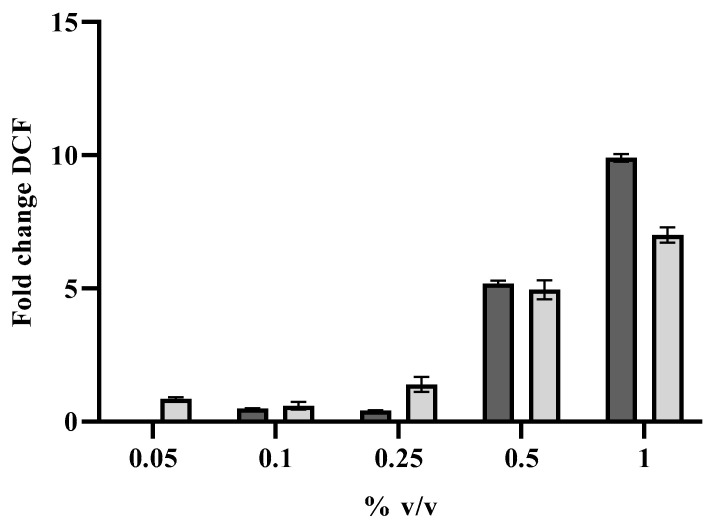
Increase in intracellular ROS in *C. auris* persister-derived biofilm following treatment with different concentrations of *L. angustifolia* free (dark grey) and liposome-encapsulated oil (light grey). Dichlorofluorescein (DCF) fluorescence values are normalized to biofilm biomass and expressed as DCF fold change with respect to untreated sample. Data are the mean of three independent experiments ± SD.

It is known that the exposure of EOs on *Candida* cells provokes an increase in intracellular Reactive Oxygen Species (ROS), which in turn may determine a cellular damage and eventually cell death, which in the case of sessile cells leads to their detachment. ROS increase was detected also in *C. auris* cells in the persister-derived biofilm, when treated with free or encapsulated oil for eradication, and the ROS increase was concentration-dependent (Figure 7).

**Figure 8:**
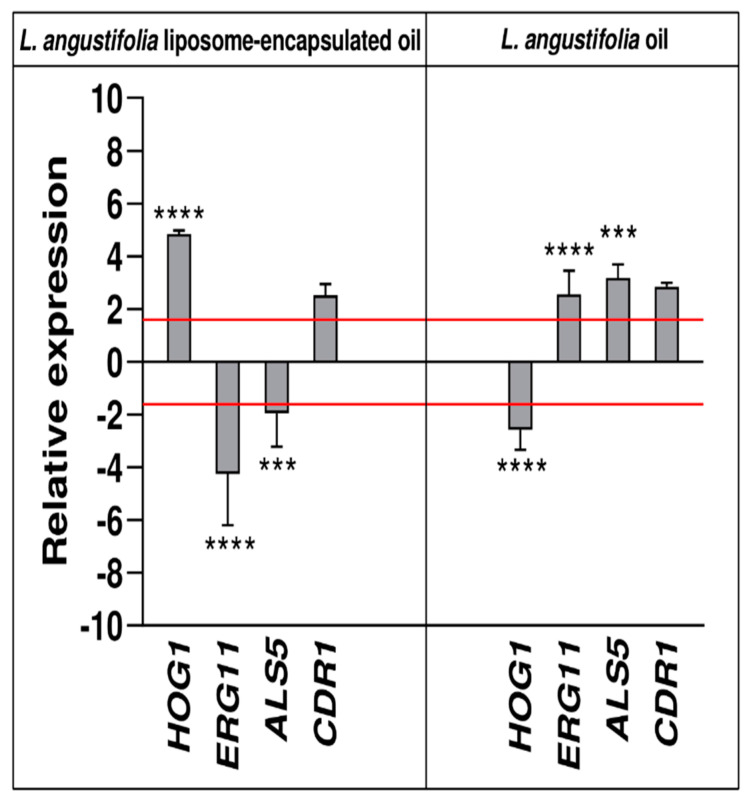
Real-time qPCR at 48 h of treatment. Histograms show the differences in the expression levels of three virulence genes, one adhesion gene, one gene involved in hyphal formation. Fold differences greater than ±1.5 (see red dotted horizontal guidelines at values of +1.5 and −1.5) were considered significant (see Table S2 for the values). A Brown–Forsythe test Sisak’s test (**** *p* < 0.0001; *** *p* < 0.001).

To elucidate the potential mechanisms by which *L. angustifolia* essential oil alone or encapsulated in a liposome (concentration of 0.05%) eradicated *C. auris* persister cells biofilms, genes expression analysis of *ALS5*, *ERG11*, *CDR1* and *HOG1* was performed using qRT-PCR (Figure 8, Table S2).

Data demonstrated that *ERG11* (*p* < 0.0001) was significantly downregulated in biofilms treated with liposome confirming that one of the mechanisms of antifungal activity could be by binding to the membrane ergosterol also in a secondary biofilm, while upregulated in persister biofilm treated with oil alone. *L. angustifolia* essential oil upregulated this gene with a 2.6-fold increase (Figure 8, Supplementary Materials: Table S2). In addition, the expression of *ALS5* was also inhibited after treatment with liposome of 2- fold, on the contrary significantly upregulated in biofilm treated with only essential oil with a 3.2-fold increase in expression levels (see also Table S2). *HOG1* gene was up-regulated (4.8-fold) and down-regulated (2.6-fold) by *L. angustifolia* liposome-encapsulated oil and *L. angustifolia* oil, respectively (*p* < 0.0001; Figure 8). Finally, *CDR1* was up-regulated by both treatments (2.5-fold and 2.8-fold increase in expression levels, respectively), as seen also in Table S2.

## 3. Discussion

Nowadays, failure in the treatment of *Candida* spp. infection has highlighted the relevant role exerted by the so-called persister cells in biofilms.

In this work, we suggest that persister cells are present also in biofilms of the emerging pathogen *C. auris*. Indeed, *C. auris* with multiple drug-resistances against prominent antifungals has occurred independently in different countries, so that a clinical alert identifying *C. auris* as an emerging “superbug” has been released [27,28]. The incoming of *C. auris* also in Italy is recent, and its spread has probably been facilitated during the SARS-CoV-2 pandemic, due to the increase in antimicrobial resistance for the unconditioned use of broad-spectrum antimicrobials [29].

Here, using an in vitro model, we showed that when a mature biofilm of *C. auris* was treated with caspofungin, a small fraction of cells tolerant to high concentrations of the antifungal was detected. *C. auris* persisters, which survived the caspofungin challenge, were able to re-colonize the support when incubated in the presence of fresh medium. This proliferation mimics the re-colonization of a surface which may follow a strong antifungal treatment, as in the case of the lock therapy [30], which utilizes prolonged instillation of a solution containing high concentrations of antimicrobial agent within an infected intravascular catheter, in an attempt to sterilize it and control bloodstream infections. Therefore, due to the survival of the tolerant persister subpopulation, in the long-term, re-population of the catheter interior may occur, thus leading to chronic infections.

Essential oils fractionally distilled from plants have drawn increased attention because of their multiple pharmacological properties such as antibacterial, antifungal, and antiviral activities. To date very few scientific papers have investigated the effect of EOs [18] and their components such as monoterpene phenols against planktonic *C. auris* [31], and none reported the effect of EOs on *C. auris* biofilms. In particular, *L. angustifolia* oil on *C. auris* has not been investigated so far.

In the *L. angustifolia* oil examined, the main components are linalyl acetate and linalool, according to the composition of this oil as reported by other authors [32]. The same authors attributed to these two components, and especially to the monoterpene linalool, the antifungal activity against *C. albicans*, though the antimicrobial effects of the oil may be the result of the synergistic action of its major and minor components [33].

Our results clearly show that low concentrations of free and liposome-encapsulated *L. angustifolia* oil are sufficient to inhibit the growth of *C. auris*. Further, and more interestingly, the same compounds were effective to eradicate biofilms formed by *C. auris* in vitro, either the biofilm developed starting form planktonic cells (primary biofilm) and that developed from persister cells. The effective concentrations remained unchanged, indirectly showing that persisters were transient phenotypic variants instead of resistant cells, giving rise to a new biofilm which responds to the antifungal treatment similarly to the primary biofilm.

*L. angustifolia* encapsulation into liposomes can be used to overcome some critical issues. The use of liposomes also provides the necessary protection against essential oil oxidation, and thus enhances the oil solubility.

Researchers have investigated the complex action modes of EOs from various perspectives. The main mechanism is based on their lipophilic character, which facilitates the access of the hydrophobic compounds to the cytoplasmic membrane, causing membrane damage, loss of intracellular substances, and finally microbial death [34]. The effect of oil or its active components on *Candida* spp. is certainly mediated by ROS generation [35]. Additionally, for *C. auris* persister cells in the biofilm, following exposure to free or encapsulated oil, we detected an increase in intracellular ROS depending on used concentrations, showing that the antifungal effect of the two compounds is mediated by ROS accumulation. It is likely that also for *C. auris*, ROS production is associated with cell damage and to the activation of the apoptotic pathway.

The impact on genes encoding for efflux pumps (*CDR1*), ergosterol biosynthesis (*ERG11*), hyphae-specific gene (*ALS5*) and stress related (*HOG1*) in the cells of the persister derived biofilm confirmed the treatment efficacy.

Indeed, in our study, the gene related to ergosterol biosynthesis, *ERG11,* was downregulated during eradication with liposome encapsulated oil, and this effect could be attributed to its cell membrane-targeting activity, while we could observe a significant increase in expression of the *ERG11* gene when the selected *L. angustifolia* oil were applied to persister-derived biofilm.

Although it had been previously shown that linalool, one of the major components of Lavandula oil, inhibited hyphal growth and biofilm formation of *C. albicans* by downregulating, between others, the expressions of *ALS3* [36], in the present study, we observed that only the gene expression of hyphae-specific *ALS5* in biofilm-persister cells treated with *L. angustifolia* liposome-encapsulated were downregulated. It appears that in persister-cells derived biofilm treated with essential oil only, other hyphal growth and biofilm formation genes had been involved to diminish its virulence.

*HOG1* was upregulated, when encapsulated oil was used, and since *HOG1* is involved in the oxidative stress response, this result strongly supports the implication of the *HOG1* pathway in the *C. auris* response to *L. angustifolia* encapsulated oil. Our results are interesting, since part of the side effects caused by antifungal compounds are due to their ability to trigger oxidative stress. When intracellular ROS is elevated, *C. auris* will initiate an effective oxidative stress response regulated by the transcription factor *HOG1*, as shown for other *Candida* spp. Regarding the expression of *CDR1,* one of the genes encoding for the efflux pump, we observed that both the application of free lavender and the encapsulated one upregulated this gene. We hypothesized that other genes correlated with resistance should be investigated.

## 4. Materials and Methods

**Figure 9:**
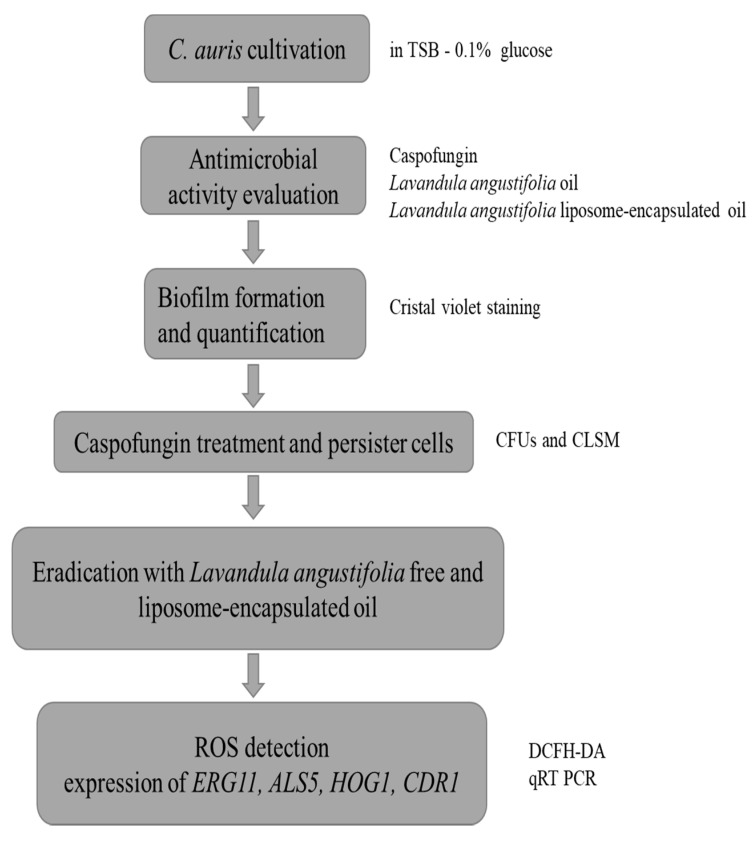
Flow chart of study design.

In Figure 9 the flow chart of the experimental design is reported.

### 4.1. Strain and Culture Condition

*Candida auris* strain DSM 21092 (DSMZ German Collection) was maintained in glycerol at −80 °C. The strain was cultured in shake flasks containing Tryptic Soy Broth (TSB) (OXOID, Basingstoke, UK) with 1% *w*/*v* glucose, starting from an overnight pre-culture prepared in the same medium. Shake flasks were incubated at 37 °C, 200 rpm.

### 4.2. GC-MS Analysis of L. angustifolia Oil

Composition of *L. angustifolia* oil (Natura Zen Srl, Italy) (Lot n. 0281-4392105-2023/2; expiry date 12/2023) was determined by GC-MS analysis performed with an Agilent 6850 GC (Milan, Italy), equipped with an HP-5MS capillary column (5% phenyl methyl poly siloxane stationary phase), coupled to an Agilent 5973 Inert MS detector operated in the full scan mode (*m*/*z* 18–550) at a frequency of 3.9 Hz and with the EI ion source and quadrupole mass filter temperatures kept, respectively, at 230 °C and 200 °C. Helium was used as carrier gas at a flow rate of 1 mL·min^−1^. The injector temperature was 180 °C and the temperature ramp raised the column temperature from 50 °C to 240 °C: 50 °C for 5 min; 5 °C·min^−1^ until reaching 180 °C; and 10 °C·min^−1^ until reaching 240 °C. The solvent delay was 3 min. Compounds were identified by comparing their EI mass spectra at 70 eV with spectra of known substances present in the NIST 14 mass spectral library (NIST 14, https://www.nist.gov/srd/nist-standard-reference-database-1a (accessed on 3 November 2021)). Moreover, the identification was supported by Kovats retention index (RI) calculated for each analyte by the Kovats equation, using the standard *n*-alkane mixture in the range C7-C40 (Sigma-Aldrich, Saint Louis, MO, USA).

The relative percentage of each compound is defined by the area of selected peak divided for the sum of the peak area.

### 4.3. Preparation of Liposome-Encapsulated L. angustifolia Oil

L-α-phosphatidylcholine (PC) and cholesterol (Chol) were purchased from Avanti Polar Lipids (Alabaster, AL, USA).

Small unilamellar vesicles (SUV) consisting of PC/Chol (70/30 mol/mol) were prepared. The liposomes were prepared at a total lipid concentration of 0.1 mM, mixed aggregates of PC, Chol and *L. angustifolia* oil were dissolved in chloroform and subsequently the solvent was evaporated under a stream of nitrogen gas. Appropriate amounts of *L. angustifolia*, in particular 20 µL were added to 200 µL of liposomes, so that oil in the liposome formulation had a concentration of 10% *v*/*v*. Following, lipid films were hydrated with water for 1 h. The lipid suspension was freeze–thawed 6 times, Liposomes were obtained sonicating the solution for 40 min. Unloaded drugs were removed by the Sephadex G50 column to purify the final formulation and evaluate the efficiency of encapsulation. Dynamic light scattering (DLS) measurements were made using Zetasizer Nano-ZS (Malvern In-struments, Worcestershire, UK), to check the Zeta potential and the size (Table 2). The analysis was performed with He–Ne laser 4 mW operating at 633 nm at scattering angle fixed at 173° and at 25 °C. The results were determined three times for each sample and each measurement was performed at least in triplicate.

### 4.4. Determination of Caspofungin Minimum Inhibitory Concentration

The minimum inhibitory concentrations (MIC) of caspofungin (Sigma-Aldrich Co, St. Louis, MO, USA) against *C. auris* were determined with a broth microdilution method, as described by CLSI-M27-A3 [37] with some modifications. Concentrations of caspofungin from 0.1 to 5 μg mL^−1^ were added to wells of a 96-well microplate containing the microorganism in TSB with 1% *w*/*v* glucose. The plate was incubated at 37 °C for 24 h. MIC value was determined as the lowest concentration inhibiting fungal growth at 590 nm using a microplate reader (Synergy H4; BioTek Instruments, Agilent Technologies, Winooski VT 05404 USA).

### 4.5. Effect of L. aungustifolia Oil and Liposome-Encapsulated Oil on Planktonic C. auris Cells

The effect of both *L. angustifolia* oil and liposome-encapsulated oil on planktonic cells of *C. auris* was evaluated incubating fungal cultures in shake flasks containing TSB medium with 1% *w*/*v* glucose, in the presence of different oil or liposome-encapsulated oil concentrations, ranging from 0.005 to 0.5% *v*/*v*. Stock solutions of the oil were prepared diluting it in 0.1% *v*/*v* Tween 80; stock solutions of the liposome-encapsulated oil were prepared in distilled water. The cultures were inoculated with proper aliquots of an overnight pre-culture prepared in the same conditions. The flasks were incubated at 37 °C for 24 h at 200 rpm. Cell growth was monitored spectrophotometrically at OD_590_ and growth reduction expressed as percentage of the control.

### 4.6. Biofilm Formation and Quantification

*C. auris* cells used to start biofilm formation were obtained collecting cells in mid-exponential phase from shake flask cultures in TSB with glucose 1% and re-suspending them in RPMI 1640 (*w*/*o* glutamine) (Lonza, Switzerland) to 1 × 10^6^ CFU mL^−1^. Aliquotes of 100 μL of cell suspensions were loaded into a polystyrene 96-well microplate and incubated at 37 °C for 24 or 48 h. Wells were then washed three times with 200 μL PBS to remove non-adherent cells.

Quantification of the biofilm biomass was performed by crystal violet staining, as previously described [33]. To quantify the biofilm formation, the optical density cut off value (ODc) was calculated as the three standard deviations above the mean OD of the negative. Final OD value of the tested strain was interpreted as negative (OD ≤ ODc), weak (ODc ≤ OD ≤ 2ODc), moderate (2ODc < OD ≤ 4ODc), or strong (4ODc < OD) biofilm former [38].

### 4.7. Detection of Persisters Cells

The quantification of persister cells in *C. auris* biofilm was performed as previously described [11], with some modifications. Briefly, the 48 h *C. auris* biofilm, grown as previously described, was challenged with caspofungin buffered to pH 7 with 0.165 M morpholinopropanesulfonic acid (MOPS, Sigma-Aldrich Co, St. Louis, MO, USA) at concentrations ranging from 5 to 200 μg mL^−1^ for 24 h at 37 °C in RPMI medium. Caspofungin stock solution at concentration of 500 mg mL^−1^ was prepared in DMSO 1.3% *v*/*v*. Preliminarly, it was shown that DMSO was not toxic against *C. auris* up to concentrations of 5% *v*/*v* (data not shown).

Biofilms were abundantly washed with PBS, then disrupted by scraping and vortexed vigorously for 30 s before serial dilution and plating on YPD (1% yeast extract, 2% bactopeptone, 2% glucose) agar medium. Plates were incubated at 37 °C for 48 h. The number of surviving cells (persisters) was expressed as CFU per well.

### 4.8. Development of Persister-Derived Biofilm of C. auris

The small number of cells not susceptible to caspofungin treatment (persisters) in the primary 48 h biofilm were the inoculum of a new biofilm (persister-derived biofilm), which developed in situ in the 96-well microplate.

Therefore, to obtain the persister-derived biofilm, following the caspofungin treatment of the primary biofilm, the wells were abundantly washed with PBS, and incubation was allowed to continue for 12 d during which 200 μL YPD medium were added to each well every 24 h. Biofilm mass was estimated every 48 h by CFU assay, as described above.

### 4.9. Eradication of Biofilm with Free Liposome-Encapsulated L. angustifolia Oil

The eradication activity of *L. angustifolia* oil, fand liposome-encapsulated oil, was evaluated exposing *C. auris* primary and persister-derived biofilms for 24 h to different concentrations of each of the two compounds ranging from 0.05 to 1% *v*/*v*, in RPMI medium and quantified the residual biomass by crystal violet assay. The percentage of eradication was calculated as biofilm reduction % = (OD_570_ control − OD_570_ sample/OD_570_ control) × 100, where OD_570_ control and OD_570_ sample corresponded to the untreated and treated biofilm, respectively.

### 4.10. Determination of iROS in Persisters-Derived Biofilms of C. auris

Intracellular ROS levels were investigated using the fluorescent dye dichlorodihydrofluorescein diacetate (DCFH-DA) (Sigma-Aldrich Co, St. Louis, MO, USA). To persister-derived biofilms of *C.auris*, developed in the wells of the microplate and treated with different concentration of *L. angustifolia* free and liposome-encapsulated oil, 10 μM 2′,7′-dichlorofluorescein diacetate (DCFH-DA) in PBS was added. Biofilms were Incubated for 1 h at 37 °C, then fluorescence intensities (excitation and emission of 488 and 540 nm, respectively) were measured with a microtiter plate reader (Synergy H4; BioTek Instruments, Inc.) Fluorescence values were normalized to biofilm biomass, as determined by crystal violet staining.

### 4.11. Gene Expression in C. auris Persister-Derived Biofilms Treated with L. angustifolia Free and Liposome-Encapsulated Oil

The effect of *L. angustifolia* free and liposome-encapsulated oil on the expression of *ERG 11*, *ALS 5*, *HOG 1* and *CDR1* (Table S1) [39,40] in persister-derived biofilms of *C. auris* was analysed using qRT—PCR. Briefly, persister-derived biofilms were treated with 0.05% *v*/*v* oil or liposome-encapsulated oil for 24 h at 37 °C. Then, the biofilms were scraped from the polystyrene surface, and collected by centrifugation (3000× *g*, 5 min). The pellets were used to extract total RNA using the Direct-zolTM RNA Miniprep Plus Kit (ZYMO RESEARCH). The purity and concentration of the extracted RNA were verified using Nanodrop spectrophotometer 2000 (Thermo Scientific Inc., Waltham, MA USA). A total of 1000 ng of extracted RNA was retrotranscribed with an iScript™ cDNA Synthesis kit (Bio-Rad, Milan, Italy) and it was used as a template in a reaction containing primer (0.3 mM) and 1× SensiFASTTM SYBR Green master mix (Meridiana Bioline, Aurogene, Rome, Italy). PCR amplifications were performed in a AriaMx Real-Time PCR instrument (Agilent Technologies, Inc., Santa Clara, CA, USA), according to the manufacturer’s instructions. Fluorescence was measured using Agilent Aria 1.7 software (Agilent Technologies, Inc.). The expression of gene was analyzed and normalized against *ACT1* gene using REST software (Relative Expression Software Tool, Weihenstephan, Germany, version 1.9.12) based on the Pfaffl method [41,42]. Relative expression ratios greater than ±1.5 were considered significant. A Brown–Forsythe test was applied, followed by Tukey`s post hoc test.

### 4.12. Confocal Laser Scanning Microscopy

The occurrence of persisters in *C. auris* biofilm was also evaluated by Confocal Laser Scanning Microscopy (CLSM). The biofilm was allowed to form for 48 h on NuncTM Lab-Tek^®^ 8-well Chamber Slides (n° 177445; 209 Thermo Scientific, Ottawa, ON, Canada) and treated with 200 µg mL^−1^ caspofungin. Then, it was thoroughly washed with PBS and stained with 5 μM SYTO9 (ThermoFisher Scientific MA, USA) that penetrates both viable and nonviable cells, followed by 20 μM propidium iodide, a non-vital nuclear stain commonly used for identifying dead cells, for 15 min at room temperature, in the dark. Observation was performed with Zeiss LSM700 microscope at 20× magnification.

### 4.13. Statistical Analysis

Statistical analyses were performed using GraphPad Prism Software (version 8.02 for Windows, GraphPad Software, La Jolla, CA, USA, www.graphpad.com (accessed on 6 November 2021)). Data were reported as the mean ± standard deviation obtained from three independent experiments. Data of development of a new biofilm from persisters and of effect of *L. angustifolia* free and liposome-encapsulated oil on planktonic cells were analysed by one-way ANOVA followed by Holm–Sidak’s test (*p* < 0.05); data of eradication were analysed by two-way ANOVA followed by Tukay’s test for the difference between the groups.

## 5. Conclusions

Our results show that persister cells tolerant to caspofungin are present in a biofilm of *C. auris*. Persisters can progressively re-colonize the support, giving rise to a new persister-derived biofilm.

*L. angustifolia* oil shows antifungal and antibiofilm properties in the two formulations tested towards *C. auris*, being effective also against persister-derived biofilm.

Overall, the results indicate the possibility of extending the use of EOs, confirming the great potential represented by the use of alternative compounds to combat emergent fungal pathogen infections.

## Figures and Tables

**Table 1:** Chemical composition of *Lavandula angustifolia* essential oils. RI represents Kovats retention index.

Compound	RI	Peak Area (%)
β-Myrcene	892	0.14
1,8-Cineole	932	1.20
β-Pinene	946	1.29
trans-Ocimene	954	0.27
Linalool	1034	39.31
Camphor	1059	0.11
Isoborneol	1078	0.95
4-Terpineol	1091	1.40
Cis-β-Tepineol	1100	0.14
α-Terpineol	1107	0.08
Linalyl acetate	1180	48.45
Lavandulyl acetate	1207	2.17
Geraniol acetate	1292	0.45
Caryophyllene	1335	2.01
β-Farnesene	1366	1.77
β-Cubebene	1390	0.07
Caryophyllene oxide	1494	0.21

**Table 2:** Mean diameter, PDI and Zeta potential of liposome-encapsulated *L. angustifolia* oil.

Sample	Mean Diameter (nm)	Mean PDI	Mean Zeta Potential (mV)
Liposome-encapsulated Oil	177.2 ± 11.49	0.295 ± 0.053	−3.73 ± 0.099

## Supplementary Materials

The following are available online at www.mdpi.com/article/10.3390/antibiotics11010026/s1, Table S1: Name, acronym, sequence and references of used primer for *RT-qPCR*; Table S2: Data of expression levels in *C. auris* biofilm.
