# Supplementary material for: Activity of Free and Liposome-Encapsulated Essential Oil from Lavandula angustifolia against Persister-Derived Biofilm of Candida auris
Source: Antibiotics (Basel). 2021 Dec 27;11(1):26. doi: 10.3390/antibiotics11010026 (PMC8772840; doi:10.3390/antibiotics11010026)
Supplement: Supplementary file 1 [file antibiotics-11-00026-s001.zip › antibiotics-1492592-supplementary.pdf]

antibiotics

Article

Activity of Free and Liposome-Encapsulated Essential Oil from *Lavandula angustifolia* against Persister-Derived Biofilm of *Candida auris*

Elisabetta de Alteriis [1], Angela Maione [1], Annarita Falanga [2], Rosa Bellavita [3], Stefania Galdiero [3], Luisa Albarano [1], Maria Michela Salvatore [4,5], Emilia Galdiero [1,*] and Marco Guida [1]

Table S1. Name, acronym, sequence and references of used primer for RT-qPCR.

Gene names	*Acronym*	Primer name	Sequence (5'→3')	Reference
Agglutinin like-sequence 5	*ALS5*	C.auris_ALS5_F	CCTTCTGGATCGGACACAGT	[29]
		C.auris_ALS5_R	AGTTGTGGTGGAGGAACCAG	
Genes encoding efflux pumps	*CDR1*	C.auris_CDR1_F	GAAATCTTGCACTTCCAGCCC	[39]
		C.auris_CDR1_R	CATCAAGCAAGTAGCCACCG	
Ergosterol biosynthesis enzyme	*ERG11*	C.auris_ERG11_F	GTGCCCATCGTCTACAACCT	[39]
		C.auris_ERG11_R	TCTCCCACTCGATTTCTGCT	
High-osmolarity glycerol1	*HOG1*	C.auris_HOG1_F	GACTTGTGGTCTGTGGGTTG	[40]
		C.auris_HOG1_R	ACATCAGCAGGAGGTGAGC	
Actin	*actin*	C.auris_actin_F	GAAGGAGATCACTGCTTTAGCC	[39]
		C.auris_actin_R	GAGCCACCAATCCACACAG	

Table S2. Data of expression levels in *C. auris* biofilm.

	L. angustifolia liposome-encapsulated oil	*L. angustifolia* oil
HOG1	4,84	-2,58
ERG11	-4,26	2,56
ALS5	-1,95	3,18
CDR1	2,52	2,85
